# A Multi-Institutional Randomized Controlled Trial to Investigate Whether Zoledronate Prevents Bone Loss After Discontinuation of Denosumab: The Study Protocol of Denosumab Sequential Therapy (DST) Trial

**DOI:** 10.3389/fmed.2021.717168

**Published:** 2021-09-08

**Authors:** Chia-Che Lee, Chen-Yu Wang, Chih-Chien Hung, Chuan-Ching Huang, Chung-Yi Li, Hsuan-Yu Chen, Yun-Liang Chang, Wo-Jan Tseng, Ting-Ming Wang, Rong-Sen Yang, Tze-Hong Wong, Shau-Huai Fu

**Affiliations:** ^1^Department of Orthopedic Surgery, National Taiwan University Hospital, Taipei, Taiwan; ^2^School of Pharmacy, College of Medicine, National Taiwan University, Taipei, Taiwan; ^3^Graduate Institute of Clinical Pharmacy, College of Medicine, National Taiwan University, Taipei, Taiwan; ^4^Department of Pharmacy, National Taiwan University Hospital Yun-Lin Branch, Douliu, Taiwan; ^5^Department of Orthopedics, National Taiwan University Hospital Yun-Lin Branch, Douliu, Taiwan; ^6^Department of Public Health, College of Medicine, National Cheng Kung University, Tainan, Taiwan; ^7^Department of Public Health, College of Public Health, China Medical University, Taichung, Taiwan; ^8^Department of Healthcare Administration, College of Medical and Health Science, Asia University, Taichung, Taiwan; ^9^Department of Orthopedics, Hsin-Chu Branch, Hsin-Chu, Taiwan; ^10^Department of Biological Science and Technology, National Chiao Tung University, Hsin-Chu, Taiwan

**Keywords:** denosumab, rebound effect, osteoporosis, zoledronate, bone loss, bone mineral density

## Abstract

**Background:** Though denosumab is an effective treatment for osteoporosis, the rebound effect after discontinuation has drawn investigators' attention. It includes a dramatic loss of gained bone mineral density (BMD) and an increased risk of vertebral fractures. This prospective multi-institutional randomized controlled trial aims to investigate whether zoledronate prevents loss of BMD after discontinuation of denosumab. The trial was registered as Denosumab Sequential Therapy (DST) trial in March 2019 at clinicaltrials.gov, with the identifier NCT03868033.

**Methods:** The study is conducted at National Taiwan University Hospital and its branches. Patients who have continuously received denosumab treatment for two or more years are surveyed for eligibility. Baseline characteristics and questionnaires of life quality are recorded after recruitment. BMD, circulating levels of bone turnover markers (BTMs), including serum N-terminal propeptide of type 1 collagen (P1NP) and C-terminal telopeptide (CTX), are checked before the stratified randomization to 4 groups. Biological sex and the T-scores are used to create 4 strata. The participants in group 1 adhere to regular denosumab therapy for another 2 years. All the other patients receive on-time zoledronate treatment in the first year. The participants in group 2, 3, and 4 have on-time denosumab, on-time zoledronate and drug holiday in the second year, respectively. BMDs are checked annually. Pre-scheduled checkpoints of BTMs are also arranged. For patient safety, rescue treatment with another injection of zoledronate will be applied to the patients on drug holiday if the CTX levels raise above the pre-specified threshold, 0.573 ng/mL for women and 0.584 ng/mL for men. The primary outcomes are the percentage changes of BMDs in lumbar spine, total hip and femoral neck. The secondary outcomes include the changes of serum level of the BTMs, new osteoporotic fractures, extra zoledronate injections needed in group 4 and the differences of quality of life.

**Discussion:** We aim to provide evidence whether zoledronate prevents bone loss after denosumab cessation. To our knowledge, the study has the largest sample size. No other randomized controlled study included all the three different treatment strategies and a positive control. It is also the first associated randomized controlled trial outside Europe.

## Introduction

Denosumab (Dmab), a monoclonal antibody against the receptor activator of nuclear factor kappa-light-chain-enhancer of activated B cells ligand (RANKL), is an effective anti-resorptive agent to treat patients with osteoporosis (1, 2). The rebound effect after discontinuation of Dmab treatment has drawn investigators' attention in recent years. The rebound effect includes a complete or near-complete loss of gained bone mineral density (BMD), and an increased risk of vertebral fractures (3–6). After cessation of Dmab treatment, the serum levels of bone turnover markers (BTM) raise rapidly in 3 months and return to baseline about 24 months later (7). The BMD loss may occur with the increased rate of bone turnover. Bone et al. reported total hip BMD would lose about 4% within 1 year after the withdrawal from 2-year Dmab treatment (7). For the patients who were treated with 1-year zoledronate (ZOL) and discontinued the treatment in the second year, the total hip BMD loss would be about 1.7% (8).

Meanwhile, vertebral fractures after discontinuation of Dmab were observed in patients receiving two or more doses of Dmab. The vertebral fractures tended to be multi-level around the thoracolumbar junction (4, 6). Ferrari reported 1–10% of the patients with Dmab cessation may have vertebral fractures (9). Compared with patients who received on-time Dmab injection therapy, those delayed a dose by more than 16 weeks were associated with increased risks for vertebral fractures (10). Our nationwide population-based cohort study also showed discontinuation of Dmab resulted in an increased risk of major osteoporotic and vertebral fractures. The increased risk tended to reveal within 1 year after discontinuation and the risk was greater among the patients with longer duration of Dmab treatment (11). In addition to vertebral fractures, higher incidences of major osteoporotic fractures and hip fractures were also observed in the following years of Dmab withdrawal (12).

The open-label multi-institutional randomized controlled trial aims to investigate whether ZOL treatment at 6 months after previous Dmab administration prevents bone loss in patients who have received Dmab for two or more years. Moreover, three different treatment strategies over 2 years for BMD preservation are also investigated with a positive control group adherent to continuous Dmab treatment every 6 months.

## Methods and Analysis

### Study Subjects and Sample Size Calculation

Post-menopausal women and men aged 50 years or older, regularly treated with Dmab every 6 months for two or more years, are evaluated for eligibility. The criteria are listed in Table 1. The patients are recruited at NTUH, NTUH Hsin-Chu Branch, and NTUH Yunlin Branch. Under the condition of 90% power and a two-sided error α probability of 0.05 with a 3.27% standard deviation (SD) (8), at least 19 patients are considered necessary in each group. Take the potential dropouts into account, the estimated sample size is around 25 in each group. Totally 100 participants are estimated to be adequate to complete the study.

**Table 1:** Inclusion and exclusion criteria.

**Inclusion criteria**	**Exclusion criteria**
Post-menopausal women or men ≥ 50 years old, regularly received Dmab for at least 2 years	1. Patients had ever used antiosteoporosis medications other than Dmab 2. Estimated glomerular filtration rate <35 ml/min. 3. Malignancy 4. Continuous steroid treatment, hormone therapy or other medical treatment affecting bone metabolism 5. Secondary osteoporosis 6. Metabolic bone diseases 7. Contraindications to ZOL 8. Patients older than 80 years old 9. Hypocalcemia

*Dmab, denosumab; ZOL, zoledronate*.

### Data Collection and the Stratified Randomization

After the acquirement of written informed consents, the baseline demographic characteristics of the recruited participants are recorded, including age, sex, body height, body weight, body mass index (BMI), history of previous doses of Dmab administration, adverse effects of Dmab, past histories of fractures, comorbidities, fracture risk assessed by Fracture Risk Assessment Tool (FRAX), histories of falls and dental conditions. Baseline BMD in spine, total hip and femoral neck regions are checked as well as baseline laboratory tests, including serum level of creatinine, serum N-terminal propeptide of type 1 collagen (P1NP), C-terminal telopeptide (CTX). The participants are also interviewed for baseline health-related quality of life through the 5-level EQ-5D version (EQ-5D-5L) (13) and World Health Organization Quality of Life–BREF (WHOQOL-BREF) questionnaires (14, 15). Study data are collected and managed using the Research Electronic Data Capture (REDCap) tools hosted at National Taiwan University Hospital and its branches (16). The participants are stratified by biological sex and the lowest T-score in total hip, femoral neck and spine region, into 4 strata. Then the stratified participants are randomly allocated via a computer-generated sequence hidden from investigators. The distribution of the enrolled cases to the four groups from the four strata are shown in Table 2. The accesses to the data recorded on the Redcap tools are allowed only for groups members in charge of data analysis.

**Table 2:** The results of randomization to the four groups from the four Strata.

**Stratification by Sex**	Stratification by T-score^*^	**Group 1**	**Group 2**	**Group 3**	**Group 4**	**Case numbers in each Stratum**
Female	T-score>−2.5	10	10	11	10	41
	T-score ≤ −2.5	13	13	14	14	54
Male	T-score>−2.5	1	1	0	1	3
	T-score ≤ −2.5	1	1	0	1	3
	Case numbers in each group	25	25	25	26	Total case numbers = 101

* *The representative T-score is the lowest value measured in lumbar spine, femoral neck, or total hip region of each participant*.

### Study Design and Intervention Methods

**Figure 1:**
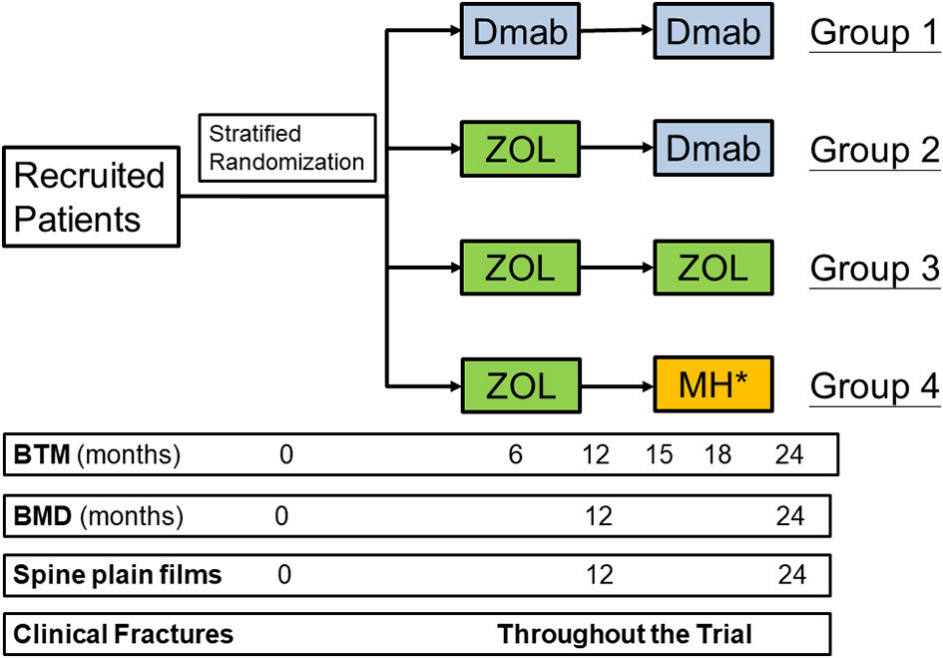
The flowchart of the study. Dmab, denosumab; ZOL, zoledronate; MH, monitored drug holiday; BTM, bone turnover markers; BMD, bone mineral density. *An extra ZOL injection will be given once the C-terminal telopeptide levels raise above the pre-specified threshold, 0.573 ng/mL in post-menopausal women and 0.584 ng/mL in men, respectively.

It is a 2-year prospective, multi-institutional, randomized controlled clinical trial. The study flowchart is shown in Figure 1. During the 2-year study period, the patients in group 1 continuously receive Dmab treatment once every 6 months for 2 years. Group 1 is regarded as the positive control group. The patients in the other three groups receive on-time ZOL, 6 months after last Dmab treatment, at the 1st year. At the 2nd year of the study, patients in group 2 switch back to have on-time Dmab treatment once every 6 months, 1 year after previous ZOL treatment. Patients in group 3 have on-time ZOL therapy in the 2nd year, while the patients in group 4 start to have drug holiday in the 2nd year. Spine, total hip and femoral neck BMDs are checked annually. Serum levels of P1NP and CTX are checked at baseline, 6, 12, 15, 18, and 24 months after the randomized allocation. Once the CTX level elevates above the pre-defined level in group 4 patients, an extra dose of ZOL will be given. For the safety of the participants, we use relatively strict and low threshold level of CTX, 0.573 ng/mL for post-menopausal women and 0.584 ng/mL in men, respectively (17–23). The events of morphologic vertebral fractures, clinical vertebral fractures and other osteoporotic fractures are confirmed by radiograph annually and whenever necessary by physician's decision. The adverse drug reactions, observed by research members or reported by the patients, are recorded. Life quality questionnaires are acquired every 6 months. We provide instruction for all participants to acquire at least 800 international unit of vitamin D3 and 1,000 mg of calcium daily.

### Primary and Secondary Outcomes

The primary outcomes are the percentage changes of BMDs in lumbar spine (LS), total hip (TH) and femoral neck (FN) among the study groups. The secondary outcomes include morphological and clinical osteoporotic vertebral fractures, other osteoporotic fractures, differences of the BTMs, extra zoledronate injections needed in group 4 and the longitudinal changes of the questionnaires-based life quality.

In the first year, we try to explore the extent of bone loss after drug switch. The percentage changes of the BMDs of the participants in group 1 are compared with those of the participants in the other groups who are treatment with ZOL in the first year. We will also investigate the factors related to significant bone loss after transition from Dmab to ZOL.

After final follow-up of the second year, we will compare the percentage changes of BMDs in Group 4 with historical negative control. Furthermore, the comparison of percentage changes of the BMDs among these four groups will be completed. The differences of circulating BTM changes among the four groups and the changes of life quality will also be investigated.

### Data Analysis

Intention-to-treat analysis will be performed. For the primary outcomes, Shapiro-Wilk test will be used to exam the normality. Normally distributed continuous data will be evaluated via one-way analysis of variance (ANOVA). Otherwise, the Kruskal-Wallis test will be applied. To detect factors related to significant bone loss after drug switch, we define significant bone loss as more than 5 % BMD loss in LS or more than 4% BMD loss in TH region according to the literature (24). We will also evaluate the associations between significant bone loss and potentially important prognostic factors, including age, sex, BMI, previous fracture history, FRAX, Dmab duration, baseline CTX level, baseline P1NP level, institution, baseline BMD in LS, FN and TH regions by univariate logistic analysis. Relevant covariates will be further included into the multivariate logistic regression analysis to identify the factors accounting for significant bone loss.

For the secondary outcomes, Fisher's exact test and chi-squared test are used to determine whether categorical data from different groups are independent. Depending on normality, one-way ANOVA or Kruskal-Wallis test will be applied for numerical data. Events of osteoporotic fractures, vertebral fractures and adverse reactions will be reported.

## Discussion

Real-world data showed that the compliance of continuous use, or the “persistence,” of Dmab ranged from 65.8 to 88% in the first year and decreased to be around 41.2–75% in the second year (25–28). During the era of COVID-19 pandemic, the persistence may drop further. Solid evidence for effective sequential therapy of osteoporosis to prevent bone loss after Dmab discontinuation is required.

Bisphoshonates (BPs) may, at least partially, preserve the gained bone mass and decrease the risk of vertebral fractures (29). There were two associated single-institutional randomized controlled trials. The Greek study group compared the treatment effect of single dose of zoledronic acid (ZOL) with two doses of Dmab, followed by direct drug holiday in women who reached non-osteoporotic BMD level. A single dose of ZOL was effective to prevent bone loss in most patients with low vertebral fracture risk at 2 years following drug switch. But three out of 27 participants still experienced BMD decrease greater than the least significant change. The bone loss was deemed to be caused by not-yet-defined intrinsic factors (30). The BTM levels elevated within 1 year after drug switch, suggesting that further cautious survey was necessary since further bone loss was possible (31). The randomized trial by the Danish group showed inevitable BMD loss in post-menopausal women and men above 50 years with osteopenia after ZOL treatment following Dmab cessation from long-term denosumab treatment for 4.6 ± 1.6 years. The bone loss corresponded to 0.25 to 0.5 standard deviation of gained bone mass, irrespective of the 6-, 9-month, or observational treatment strategy. On-time treatment with ZOL seemed to be the most attractive strategy among the investigated options in the study (32). Although a single dose of ZOL may be helpful, the individual setting for sequential therapy may vary widely regarding the baseline fracture risk, bone turnover rate, duration of Dmab treatment and other factors. Further randomized controlled trial was deemed to be particularly necessary (9).

### Strengths

To the best of our knowledge, this is the first “multi-institutional” study among the randomized controlled trials about subsequent treatment after Dmab discontinuation. The current trial may also include the largest sample size among the randomized controlled trials. The other strengths of the study are as the following. Firstly, we include both men and women with osteoporosis or osteopenia to evaluate therapeutic effects in different disease status. By these means we may improve the external validity of the study. Through stratified randomization, the biological characteristics, and the severity of osteoporosis along with possible confounding factors are expectantly to be equally distributed among four groups.

Secondly, we have four study groups with different treatment strategies. Group 1 stands as the positive control. In the first year, we may illuminate the extent of bone loss in LS, TH and FN regions after drug switch by comparing the percentage changes of the BMDs between the patients treated with Dmab and those with ZOL. The factors associated with significant bone loss after drug switch may provide important clinical implications. Group 2 will show the percentage changes of BMD after double drug switch, which has not yet been investigated previously. Group 3 will exhibit the effect of two consecutive ZOL injection on BMD and BTM levels. We will assess BMD and BTM levels changes after 1-year drug holiday following drug switch in group 4 participants. Comparison between group 3 and 4 may provide crucial information. Presumably two injections of ZOL may preserve more bone than one injection. The changes of BTM levels 1 year after drug transition and how it responds to the second injection of ZOL, will be observed. The percentage changes of BMDs in Group 4 will be compared with historical negative control due to ethical concerns. Thirdly, we have regular BTM checkpoints to show the chronological changes with different treatment strategies. Finally, we are the first Asian RCT study concerning Dmab sequential therapy.

### Limitations

To begin with, we will not be able to evaluate the therapeutic responses of patients in different timelines of ZOL treatment, as done by the Danish group. However, according to Sølling et al., on-time treatment may be the most effective and attractive option (32). Secondly, according to the database survey by the investigators, we have fewer male patients having long-term Dmab treatment. The male population in the recruited participants may drop even further, as shown in Table 2. This corresponds to the real-world situation in osteoporosis treatment (33). As mentioned above, the potential confounding factors may be reduced by the stratified randomization. Furthermore, due to ethical concerns, we do not design a group with direct drug holiday after Dmab cessation as the negative control. Historical control is applied instead. Thirdly, the dual-energy x-ray absorptiometry (DXA) devices are not unified among the institutions. This is commonly seen among the multi-institutional studies like, for example, the FREEDOM trial (34). We use General Electric Lunar Prodigy (General Electric Healthcare), Stratos DR (Diagnostic Medical Systems-Imaging) in NTUH, Stratos DR in NTUH Hsin-Chu Branch and Hologic (Hologic Inc.) in NTUH Yunlin Branch, respectively. Each participant is evaluated by only one specific type of device throughout the study. We use percentage changes of the BMDs as the outcome measures to eliminate the potential bias from the absolute values of the BMDs generated from different devices.

In summary, we aim to provide evidence to determine whether ZOL treatment prevents bone loss after Dmab cessation. We also try to determine the effectiveness of three sequential therapeutic strategies. The potential for extension of the study is preserved.

## Ethics Statement

The studies involving human participants were reviewed and approved by National Taiwan University Hospital Research Ethics Committee. The patients/participants provided their written informed consent to participate in this study.

## Author Contributions

S-HF designed the study with the help from C-YL and C-YW. C-CL, S-HF, and T-HW are the project instructor in NTUH, NTUH Yun-lin Branch, and NTUH Hsin-Chu Branch, respectively. S-HF, C-CL, and H-YC handled the research ethics. C-CL, Chi-CH, Chu-CH, H-YC, Y-LC, T-MW, W-JT, and S-HF contributed to the execution of the study. C-CL wrote this manuscript as checked by C-YW and S-HF. Supervision is provided by C-YW, C-YL, T-MW, R-SY, T-HW, and S-HF. Statistics are managed by C-YL, C-YW, and S-HF. All authors have given their final approval of the version to be published and agree to be accountable for all aspects of the work.

## Funding

This study was supported by the Research Assistantships funded by the Ministry of Science and Technology, Taiwan (grant number MOST 108-2314-B-002-100-MY3, to S-HF); National Taiwan University Hospital Yun-Lin Branch (grant number NTUHYL109.X009 & NTUHYL109.F005, to S-HF); National Taiwan University Hospital (grant number NTUH 109-031, to C-CL). The funders have no role in the study design, data collection, analysis, interpretation nor the writing of the report.

## Conflict of Interest

The authors declare that the research was conducted in the absence of any commercial or financial relationships that could be construed as a potential conflict of interest.

## Publisher's Note

All claims expressed in this article are solely those of the authors and do not necessarily represent those of their affiliated organizations, or those of the publisher, the editors and the reviewers. Any product that may be evaluated in this article, or claim that may be made by its manufacturer, is not guaranteed or endorsed by the publisher.
